# Texas State Medical Association

**Published:** 1896-05

**Authors:** P. C. Coleman

**Affiliations:** Colorado City, Texas, President


### Society Notes.

### TEXAS STATE MEDICAL ASSOCIATION.

### President's Address—Duties and Ethics of the Medical Profession.

[Dr. P. C. Coleman, of Colorado City, Texas, President.]

In obedience to the constitution of the Texas State Medical Association, which requires the president shall deliver an annual address, I am before you, ladies and gentlemen, on this occasion. From the fact you have been invited here this evening, to hear what we have to say, the inference necessarily follows, you expect to hear something which will instruct, or at least entertain you. I hope you will not be wholly disappointed. In order to allay any fears some of you may have that your patience will be taxed to listen to an address upon some abstruse, strictly medical subject, I will say now in the beginning, while I shall speak to you, of medicine and of doctors, I shall steer clear of technical phraseology. One of the masters in English literature uses this language: "Certainly it is excellent discipline for an author to feel that he must say all he has to say in the fewest possible words, or his reader is sure to skip them; and in the plainest possible words, or his reader will certainly misunderstand them. Generally, also, a downright fact may be told in a plain way; and we want downright facts at present more than anything else." I shall remember this language in speaking to you this evening.

It is no reflection on the intelligence of this cultivated audience, to ask your indulgence for a few moments to define the terms medicine and physician, as they shall be used on this occasion; in such a way we hope as shall in some degree at least, interest those whose kindly presence lends grace and encouragement to this our annual session. Medicine: "The art of preventing, curing or alleviating diseases and remedying as far as possible, the results of violence and accidents." It is evident then if this definition be true, that the science of medicine can not be a sect or school, any more than the science and art of navigation or agriculture. It is equally clear that physicians who study and practice the science and art of medicine are not allopaths, homeopaths, or electropaths. Unfortunately the general public does not realize this, and the majority of medical men are called allopaths, simply because they are not homeopaths. This is not true. Allopathy means "the employment of medicines to produce effects different from those resulting from disease. Physicians do not subscribe to a law of contraries or dissimilars (allopathy) as the homeophaths do to a law of "similars." This language was used by a gentleman recently on an occasion similar to the one which brings us together this evening, and is very true. Physician, as used, refers to the regular practitioner, who follows no clique, sect, pathy or ism.

So far as I know, there may be those present who honestly differ from us in this definition as given, and if so, we have no controversy with them, but will say, however, as a student of the history of medicine, if any great discovery, any grand achievement in the medical world, which stand like signals upon the mountain tops, have come from other source than the regular profession, we have never heard of them. This is for those who are honest in their convictions. Now, for the crowd calling themselves doctors, strangers to honesty, to honor and to every pure emotion of the heart, who for the sake of money, hesitate not at any kind of deception or the performance of crime, we will pay our respects in the "fewest, plainest," possible words. You will remember Dante in his great epic poem, Inferno, places Brutus in the fourth and last round of the ninth circle of hell; why? He betrayed his friend, guilty of ingratitude, "that which is abhorred by God and man." If Dante lived at the present day, and beheld the deeds of this crowd his genius would create for them as the only suitable place, one more circle in the fourth and last round of hell, beyond and far below the one now occupied by Brutus.

In these times we often hear of the wonderful strides in medicine, all of which is true, but we should not forget, nor affect to despise as we sometimes do, what we owe the early fathers, and those who have gone before, coming on down to our own day. In their powers of observation they were our equals if not superiors.

The profession, at all times, has been composed of a community separated from a generation, and of course partook of that generation. Medicine, physics and metaphysics, have been sisters from the beginning, and have traveled side by side, since the beginning. When philosophy explained inexplicable phenomena upon the hypotheses of witches diabolism, no wonder that physicians should resort to magic, and amulets, the king's touch, appeals to dead men and women canonized by the church as saints, and to every abomination in the animal, vegetable and mineral kingdoms. When, in the beginning of the seventeenth century, the Bishop of Chester and the Bishop of Llandaff, each was worrying himself, and his diocese in publications going to prove that there were people in the moon, and that we earthites could find means to visit them in flying machines, "large enough to carry divers men, and commodities for traffic," no wonder surgeons applied their dressings, not to the wound, but to the instrument that made it. When credulity and superstition lorded it over mankind, is it to be regarded as strange that they lorded it over the doctor portion as well? The wisest judges of the law taught from their own observation, that the wounds of a murdered man, long dead, would bleed afresh upon the approach and touch of the murderer, and the trial by fire or forcing the suspected criminal to run blindfold and barefooted along a path strewn with plates of red hot iron, the verdict being, no burn, no guilt, and vice versa, was a common expedient. Physicians were as superstitious and credulous as other people, and no more so. These are well established historical facts. Does medicine suffer by the comparison?

Such things existed until the coming of the inductive method, which completely revolutionized the face of philosophy, and the whole family of the sciences, placing medicine with the other positive knwoledges upon a sure foundation.

You are aware, doubtless, that the offices of priest and physician were originally one, and filled by the same individual. That this is true, the following old-time advertisement will sufficiently show:

"Wanted—For a family who have had bad health, a sober, steady person in the capacity of doctor, surgeon, apothecary and man mid-wife. He must occasionally act as butler, and dress hair and wigs. He will be required sometimes to read prayers, and to preach a sermon every Sunday. A good salary will be given."

If he performed faithfully and efficiently all these offices he was entitled to a most munificent salary.

Those who at this day would restrict the definition of the word "physician" to terms descriptive of a mere healer of the sick or manipulator of broken bones, have a very inadequate conception of the place and function of the profession. I have no intention of underestimating this most exigent and constant, if not most important element of our work. It is of unsummed priceless benefaction to mankind every day of every year. All over the world we are without ceasing, quieting pain, repairing injuries, restoring lost harmonies, and saving multitudes from premature death. This the most careless see. But in addition to this we are doing a work, if less apparent to the senses, of immeasurably more important bearing upon the physical, social and moral destiny of mankind. We are educating the age.

This is no doubtful assumption. Physiology, hygiene, questions of quarantine and sanitary regulations, are rapidly coming to the front in the interest of the thoughtful even among non-professional men. It is everywhere in process of recognition that health, the first of blessings, is a subject of law. The most recondite sources of the highest mathematics are taxed to supply formulæ for life averages and contingencies. And here the laity must come to us for data and direction. It is the subject we deal with most constantly and most intimately. We hear nearly every murmur of discontent, and are summoned to treat almost every lesion. The confessional of pathology—the only confessional where the truth is always told, or if not told, is known—is not shut day or night. The woes of the world come to our ears. We are the final repository of its sorrows and its sins. What a revelation of the sad side of humanity would a combined experience record of the doctors make!

Already we are called to give the verdict of science upon the most vital questions that concern and interest men. You can not have a murder trial without half a dozen medical gentlemen upon the witness stand. I am sorry for the sake of science that as a rule they coincide so perfectly. It is hardly too much to say you can not shut up a recalcitrant lunatic except upon a doctor's commitment. And once in durance all the habeas corpus in the land will not avail against his writ of ne exeat. If it be sought to deprive a man of the use and disposal of his property—a thing prohibited by our most fundamental law—it can be done under a commission of delunatico inquirendo, if the doctor says yes! If it be desirable to break the last wish and will of some mortal who has passed into silence, it can not be done if the doctor says no. So you see, my friends, that we hold the fullest hand in the game of life, and are as a class and profession the governing factors in the social activities of the times. With judges and lawyers and jurymen, jurisprudence, legislation, philosophy, and every form of social inquiry and speculation going to school to the doctors. We are, in a sense. applicable to no other class of men, educators of public opinion But all this brings responsibility, and the greatest obligation, to prepare wisely to fill this great office. It is more than judicial. It is in effect, in general results, that of judge and jury in one.

With a full appreciation, and all due respect to the progressive era in which we live, I must say, from what I have been able to learn from a study of the question, that the time when the public had the proper conception of the relation of the physician to the community existed in our country prior to the late war, owing, doubtless, largely to the character of the physicians themselves. In those days practitioners of medicine were for the most part dignified, courteous gentlemen, whose intellectual attainments and excellent judgment awarded them a high place in the esteem of the community, seldom equaled and never surpassed. There are living illustrations of the truthfulness of this statement in the persons of some who are still identified with this association; and have we not been impressed with the accuracy of this statement when brought in contact with them? Yes, I always lift my hat to the old Southern country doctor. I say Southern because my observation has been confined to this section, and I am very grateful that they did not all pass from the stage before I came. Yes, all honor to the country doctor. Time will not suffice me to even mention the names of those who have by their genius placed American medicine and surgery upon the topmost pinnacle of the science.

The following touching tribute to the country physician by a recent writer will recall to some in this audience a familiar scene in the old home in Kentucky, Tennessee, Virginia, or elsewhere: "Our country physicians have so many hardships, so many interruptions, so many annoyances, I am glad they have so many encouragements. All doors open to them. They are welcome to mansion and to cot. Little children shout when they see them coming down the road, and the aged, recognizing the step, look up and say, 'Doctor, is that you?' They stand between our families and the grave, fighting back the troops of disorder that come up from their encampment by the old river. One day there was a dreadful foreboding in our house. All hope was gone. The doctor came four times that day. The children put away their toys and walked upon tiptoe, and at the least sound said 'Hush!' How loudly the clock did tick, and how the banister creaked, though we tried to keep still. That night the doctor stayed all night. He concentrated all his skill upon the sufferer. At last the restlessness subsided into a calm, sweet slumber, and the doctor looked up and said, 'The crisis is passed.' When propped up with pillows in the easy-chair she sat and the south wind tried to blow a rose leaf into her faded cheek and the children brought flowers—the one a red clover top, the other a violet from the lawn—to the lap of the convalescent. And as we helped the old country doctor into his gig we did not notice that the step was broken, or the horse stiff in the knees, and we all realized for the first time in our life what doctors were worth. Encourage them. They deserve every kindness at our hands."

In MacLarens' inimitable book, "A Doctor of the Old School," the character of McClure is not an impossible one. Nor is it confined to the highlands of Scotland. All over this land of ours has the counterpart of McClure lived and labored in the personages of our country doctors.

Many scenes through which they passed have been at times the most dramatic the world has ever witnessed. In 1818, in the wilds of the almost primeval woods of Kentucky, there was enacted a scene, which, in its results, meant more for the gentler sex than has fallen to the lot of but few. Ephraim McDowell, the forest-born Dupuytren, with his trembling yet hardly less heroic patient, Mrs. Crawford, hand in hand ventured into the yet unexplored region of Ovarian Pathology. There was no anesthetic cup to benumb the quivering tissues, and no modern antiseptic appliance to restrain the ravage of purulent infection, no one hundred consecutive cases to point her to without a single death. The stake for both was of tremendous import. To her with no record whatever of the hazardous enterprise to cheer or comfort, it did seem like laying her body upon the altar of sacrifice. To him it was a fearful responsibility, fraught either with the reproach of her life upon his soul, or with immortal fame in rescuing her from the jaws of death. The outcome was health to her, after months of suffering, and to him a heritage of renown that will continue to brighten as the ages roll into eternity.

The world has been slow to appreciate the work done by the medical profession. By comparison the work is far beyond that of all other professions. I only refer to secular work. The ministry is not a profession, but a sacred calling. I would call your attention to the fact, in all ages we have by years of toil and patience, at last by the triumph of genius, delved from various sources those great secrets of our art which add to the comfort, security and happiness of mankind. As was done for our brethren of the legal profession, with whom we are so often compared, no infinite hand on Sinai's sacred mountain, amid "the thunderings and the lightnings and the noise of the trumpet, and the mountains smoking," delivered to us "a law and commandments which He had written that we mayest teach them." If there is upon the statute books of any government any law not based upon this revelation it fails in the accomplishment of justice, which should be the prime object of all laws.

Sir Richard Quain recently cites the great work done by Dr. Snow in saving Great Britain from epidemic cholera for the last twenty-five years, and of course for all time to come. He says: "Through saving Great Britain from cholera he has saved millions of lives. The sole reward England has conferred upon him is midnight obscurity. If he had been a soldier instead of a doctor; if he had slain his thousands instead of saving his millions, every town would have hailed him as a hero, and the nation would have honored his memory with monuments more enduring than brass." My friends, you know these words are true, and you know still further such should not be.

John Hunter, the founder of scientific surgery, with his scalpel in the dissecting room, did more for England than did Lord Nelson with shot and shell at Trafalgar. Edward Jenner, after twenty long weary years of toil, in his great discovery of vaccination preventing small-pox, did more for England than did the Duke of Wellington when he overthrew her bitterest foe, the great Napoleon, at Waterloo.

We point with pride to the fact, medicine is a progressive science. In 1886, Austin Flint was invited to deliver an address before the British Medical Association. The address was prepared, but never delivered. Before the meeting of that body, this highest type of American citizen and doctor passed beyond the river into his eternal rest. For fifty years he had been a student and practitioner of the profession he so loved and honored. In this address, referring to some of the great discoveries, which made the latter part of the last half century so famous in the history of the medical world, he uses this language: "Now suppose that whoever may be honored by an invitation to deliver an address on medicine at the annual meeting of the British Medical Association in the year 1936, should select as his theme the history of medicine for the preceding half century, is it to be doubted that the epochs belonging to the history will be found to be not less in number and in importance than those which signalized medical progress during the last half of the century ending in that date? Does not the history of medicine show an acceleration in progress, so that, judging by the past, these next fifty years will be richer in epochs than the previous half century?"

Little did the great Flint think the wonderful achievements in analytical and organic chemistry, in electricity, in bacteriology, would come before the expiration of this century; some of them we have seen, and others we are in sight of with but little doubt we soon shall see.

Now, my friends of the laity, I want to repeat the assertion, medicine has kept pace with her sister sciences from the beginning. This assertion will bear the crucial test of thorough investigation.

Whenever, in the history of the world, there appears a cluster of men that can not die, but, embalmed among the immortals, gracefully descend along the line of the ages, I find that it has pleased God that medicine should not be unrepresented. When the new star shone divinely in the heavens, the cynosure of the Eastern Magi, the subsequent center of apostles, and evangelists, the incarnation; that event around which centers the destiny of every son and daughter of Adam; here medicine was represented in the person of the classical scholar, and "beloved physician," the apostle Luke. And before, when Greece had produced such a cluster of intellectual giants, which, to repeat, would require that nature must rest for centuries, when Pindar, Aristophanes and Euripides, as poets, together with Aeschylus and Sophocles, and the philosophers, Socrates, Plato and Xenophon; the historians, Herodotus and Thucidides, and Phideas, the statuary, were contemporaries and fellow citizens. Medicine was among them, in a person greater than either, who rose higher, and shone with a diviner glory, than the most illustrious of them all, in the person of the God-like Hippocrates. When Sir Isaac Newton and John Milton assimilated the philosophy and poetry of Great Britain with the gods, medicine rose with them in a person as illustrious as either, whom mankind has stamped with immortality and placed beside the founder of his art, Thomas Sydenham, the Hipprocrates of England. And when in the New world, the first cluster of immortals appeared in the persons of Washington, Franklin, Adams, Jefferson and kindred spirits, He who fashioned them saw fit to place beside them the immortal physician, Benjamin Rush. Giving Nature time to collect her forces and coming along down the line, an exceeding brightness flashes upon our mental vision, in the persons of the great trio, Clay, Webster and Calhoun; and it pleased Him who gave them to immortality, to place medicine beside them, in the person of the illustrious Drake, the peer of either in all the elements of human greatness.

To my vision, the hand of God is as manifest in the life-work of Thomas Sydenham, John Hunter and Edward Jenner, as in the life-work of Martin Luther, John Calvin and John Wesley. Look at the work of Hunter, a man as Dr. Gross truly says: "Of extraordinary genius, one of those rare beings whom an all wise and beneficent God, at long intervals, sends into the world to astonish and enlighten mankind, and to direct the human intellect into new channels of thought and action. The sparks which were emitted by Hunter's genius kindled a flame which set the medical and scientific world on fire."

Now coming to an epoch, in the history of our own country, in which many in the audience participated, I want to refer to the greatest military genius, together with the most remarkable lieutenants, of which we have any knowledge.

If the time ever comes when the truth shall be written, this is what will be accorded Stonewall Jackson and his commanders.

I speak of these in no partisan spirit, my friends, far be it from my thoughts. I care not whether the beardless boy who sleeps in his lonely grave, left his New England home and laid down his young life following Sheridan and Grant, or whether he went from Tennessee, Virginia, or Texas, and followed Forrest, Stuart, or Hood to death and glory. As American citizens, their deeds of valor from honest convictions of duty should touch a responsive chord in every true and brave heart, and should be regarded as our common heritage. God have pity on the soul so narrow and "selfish it can find nothing to admire in the courage, devotion and heroism of his enemies."

I have said this much that no one may misapprehend the allusion to the great southern soldiers. Yes, it is true, the army which Stonewall Jackson led from Manassas to Chancellorsville was commanded by the most extraordinary military genius we have any account of in the annals of the military world. Here again we find medicine represented by one we love to honor, in all the elements of human greatness the peer of the most illustrous of them, the great surgeon, Hunter McGuire.

